# Preserved muscle oxidative capacity in COPD patients with hypoxemia treated by long term O_2_ therapy

**DOI:** 10.1007/s00421-026-06227-4

**Published:** 2026-04-06

**Authors:** Alessandra Adami, Michele Vitacca, Richard Casaburi, Beatrice Salvi, Carla Simonelli, Harry B. Rossiter, Mara Paneroni

**Affiliations:** 1 Department of Kinesiology, College of Health Sciences, University of Rhode Island, 25 W Independence Square, Kingston, RI 02881 USA; 2 Respiratory Rehabilitation Unit Lumezzane Institute, Istituti Clinici Scientifici Maugeri IRCCS, Lumezzane, BS Italy; 3 Respiratory Research Center, The Lundquist Institute for Biomedical Innovation at Harbor–UCLA Medical Center, Torrance, CA USA; 4 Division of Respiratory and Critical Care Physiology and Medicine, Harbor-UCLA Medical Center, Torrance, CA USA; 5 Cardiac Rehabilitation Unit Lumezzane Institute, Istituti Clinici Scientifici Maugeri IRCCS, Lumezzane, BS Italy

**Keywords:** Long-term oxygen therapy, Lung diffusing capacity for carbon monoxide, Chronic hypoxemia, Near-infrared spectroscopy, Skeletal muscle, Smokers

## Abstract

**Purpose:**

Long-term oxygen therapy (LTOT) is an established treatment for patients with chronic obstructive pulmonary disease (COPD) diagnosed with hypoxemia at rest or during activity. Chronic hypoxemia is a potential mediator of loss of muscle mass and oxidative capacity in COPD. In this retrospective analysis, we sought to determine whether patients with COPD receiving LTOT have impaired muscle oxidative capacity compared to those without LTOT.

**Methods:**

Personal characteristics, medical and smoking history, spirometry, diffusing capacity, use of LTOT, 6-min walk distance (6MWD), dyspnea symptoms (mMRC Dyspnea scale) were extracted from medical records. *Gastrocnemius* muscle oxidative capacity was assessed from the O_2_ consumption recovery rate constant (*k*) using near-infrared spectroscopy. Chi^2^ and t-tests assessed group differences. ANCOVA was used to compare *k*, after adjusting for known covariates (FEV_1_%predicted, age, race), in COPD patients with and without LTOT.

**Results:**

23 COPD with LTOT (LTOT) and 29 COPD (non-LTOT) were included in the analysis. The LTOT group tended to be older (p = 0.060), had worse spirometry and diffusing capacity (p < 0.023) and 6MWD (p < 0.001), but similar dyspnea (p > 0.105) compared to the non-LTOT group. After adjusting for covariates, *k* was not different between groups (LTOT, 1.06 ± 0.07 vs non-LTOT, 1.09 ± 0.26 min^−1^; p = 0.436).

**Conclusions:**

Contrary to our hypothesis, muscle oxidative capacity was not lower in LTOT-treated COPD compared to those without LTOT. Because COPD patients with LTOT had lower FEV_1_%pred and tended to be older (known correlates of *k*), our findings suggest that LTOT may protect against loss of muscle oxidative function in COPD patients with chronic hypoxemia.

**Graphical abstract:**

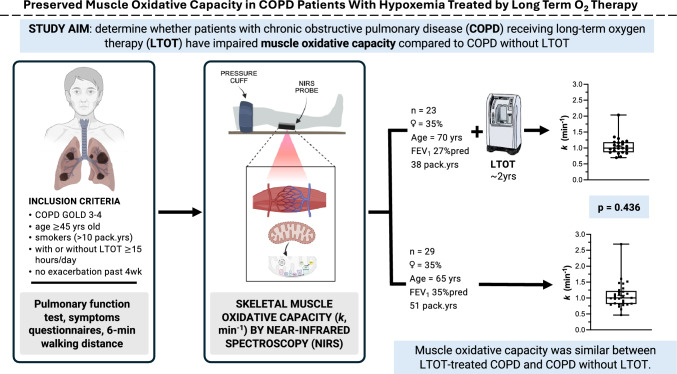

## Introduction

Chronic obstructive pulmonary disease (COPD) is one of the most prevalent causes of morbidity and disability worldwide (Kent et al. 2011), and the fourth leading cause of death globally (WHO 2021). As the disease progresses, the risk of hypoxemia increases (Casaburi and ZuWallack 2009). Approximately 7% of patients with moderate to severe COPD develop resting hypoxemia within 5 years of diagnosis (Wells et al. 2016), with a consequent reduction in quality of life, independence, and life expectancy, especially in severely obstructed patients (Kent et al. 2011). While the etiology is often unclear (Ergan and Nava 2017), intermittent or chronic hypoxemia (defined as arterial partial pressure of oxygen (PaO_2_) ≤ 55 mmHg or oxygen saturation by pulse oximetry (SpO_2_) ≤ 88% (Jacobs et al. 2020)) is proposed as a driver of skeletal muscle wasting, dysregulated muscle protein homeostasis, and mitochondrial dysfunction. Patients with COPD and nocturnal hypoxemia experience even greater muscle loss (by ~ 10–25%) compared to healthy peers, and the role of hypoxemia in mediating this loss is supported by data from in vitro and in vivo models (Attaway et al. 2024, 2023).

A substantial majority of COPD patients with moderate-to-very-severe disease manifest skeletal muscle abnormalities and dysfunction especially in the muscles of ambulation (Maltais et al. 2014), where muscle atrophy and weakness, a switch towards glycolytic, highly fatigable, type IIx MHC expressing fibers, loss of mitochondrial oxidative capacity, and increased ROS production are observed (Jakobsson and Jorfeldt 1995; Casaburi 1999; Maltais et al. 2014; Whittom et al. 1998; Adami et al. 2026). Chronic hypoxemia is suggested as a potential mediator of these effects in COPD patients (Storgaard et al. 2018; Wust and Degens 2007; Koechlin et al. 2005; Attaway et al. 2024; Stein et al. 1982), leading to impaired bioenergetic function and reduced muscle endurance. Muscle phosphocreatine breakdown during exercise in hypoxemic COPD patients is greater than in controls, and this deficit is only partially recovered (~ 20%) by restoring PaO_2_ to control levels using supplemental oxygen (Payen et al. 1993). Furthermore, chronic hypoxemia is associated with shorter quadriceps endurance time and greater muscle oxidative stress compared with non-hypoxemic COPD patients (Koechlin et al. 2005). Each of these findings is consistent with the loss of mitochondrial oxidative capacity in chronically hypoxemic COPD patients compared with non-hypoxemic COPD or healthy controls.

Long-term oxygen therapy (LTOT), defined as oxygen prescribed for $\ge$ 15 h/day (Jacobs et al. 2020), is an established treatment for COPD patients with chronic hypoxemia at rest and during activity (Storgaard et al. 2018; Raza et al. 2018). Regular use of LTOT seems to associate with reduced hospitalization and mortality rates, improved mental health and quality of life in patients with COPD but these results are inconsistent across studies (see e.g., Guell Rous 2008; Raza et al. 2018; Wells et al. 2016; Jacobs et al. 2020; Long-Term Oxygen Treatment Trial Research et al. 2016)). Neverthless, patients with daytime resting chronic hypoxemia benefit more from LTOT than those with solely nocturnal and/or exercise-induced desaturation (Long-Term Oxygen Treatment Trial Research et al. 2016). In a small study, the muscle phosphocreatine-to-total creatine ratio was significantly increased in the quadriceps of hypoxemic COPD patients (n = 4) after 8 months of LTOT compared with non-hypoxemic COPD patients (n = 4) receiving usual care (Jakobsson and Jorfeldt 1995). Together, the available data suggest that LTOT may ameliorate the muscle oxidative capacity loss observed in chronically hypoxemic COPD patients, but to confirm its benefits further investigations are warranted (Attaway et al. 2024).

In this retrospective analysis, we sought to determine muscle oxidative capacity in a large group of chronically hypoxemic COPD patients treated with LTOT compared to COPD patients without hypoxemia and LTOT prescription. We used a novel, non-invasive method based on near-infrared spectroscopy (NIRS) combined with a series of short-duration (5-to-10 s) intermittent arterial occlusions to determine the locomotor muscle oxygen consumption (mV̇O_2_) recovery rate constant (*k*) (Adami et al. 2017), which is proportional to muscle mitochondrial oxidative capacity (Wüst et al. 2013; Ryan et al. 2014). This NIRS-based protocol allows in vivo determination of muscle mitochondrial impairment in patients with chronic disease, isolated from influences of circulatory and pulmonary function (Adami and Rossiter 2018). We hypothesized that severely obstructed COPD patients with chronic hypoxemia treated by LTOT would have lower muscle *k* compared to severe COPD patients without chronic hypoxemia.

## Methods

### Study overview

This was a retrospective analysis of patients enrolled in two different studies who underwent the same evaluation of locomotor muscle oxidative capacity using NIRS. The two studies were: (1) the observational *Muscle Health Study* (Adami et al. 2026), an ancillary of COPDGene (Regan et al. 2010) (NCT00608764), that took place between 2014 and 2016 at the Respiratory Research Center at the Lundquist Institute and received approval by the Institutional Review Board of The Lundquist Institute at Harbor-UCLA Medical Center (20403-01); and (2) a randomized controlled trial (NCT04201548; CE2288) that took place at ICS Maugeri between 2019 and 2022 and received approval by local Ethics Committee (CE2288), for which only baseline (pre-intervention) data are presented here.

Participants included in this report had a confirmed diagnosis of severe or very-severe COPD (GOLD stage 3–4), with or without LTOT. The main inclusion criteria were: over 45 years of age; current or former smokers (> 10 pack-years); no exacerbation within at least 4 weeks of enrollment. The main exclusion criteria were: presence of significant disease other than COPD (defined as a disease which may influence the results of the study, such as ischemic heart disease, musculoskeletal or renal disease); active participation in pulmonary rehabilitation or participation in the past 18 months before undergoing testing. The inclusion and exclusion criteria were determined by reviewing study records, where medical and smoking history, LTOT use and prescribed medications were self reported.

Once eligibility was determined, data were extracted from study records: demographics (age, sex, race, body mass, height); medical history; concomitant medications; smoking history (ATS pack-years); presence of comorbidities; pulmonary function; use (duration in years) of LTOT prescription; dyspnea (modified Medical Research Council Dyspnea scale, mMRC); disease impact (COPD Assessment Test, CAT); functional exercise performance (6-min walking distance; 6WMD); resting arterial oxygen saturation estimated by pulse oximetry (SpO_2_) while breathing room air or breathing O_2_ using a nasal cannula at the prescribed flow rate for those prescribed LTOT; and NIRS-derived *k*, which is proportional to oxidative capacity. Fraction of inspired oxygen (FiO₂) was estimated by: FiO₂ (%) = 20 + (4 × O₂ flow rate in L/min using cannula) (Shapiro et al. 1982).

During 6WMD and NIRS testing, participants were allowed to use supplemental oxygen at the prescribed flow rate.

### Pulmonary function and arterial blood gas analysis

Spirometry was performed following American Thoracic Society guidelines (Graham et al. 2019), before and after administration of two puffs of metered dose albuterol sulfate. The absolute forced expiratory volume at 1 s (FEV_1_) and the forced vital capacity (FVC) were measured from the greatest values obtained from up to eight maximum expiratory maneuvers, where the greatest two measurements were within 150 mL.

The DL_CO_ was measured in accordance with European Respiratory Society/American Thoracic Society standards for single-breath measurements of carbon monoxide uptake in the lungs (Macintyre et al. 2005). Values were adjusted for hemoglobin concentration and altitude, and the relative (percent predicted) values were calculated using Global Lung Initiative reference equations (Miller et al. 1983).

In a sub-group of the LTOT group (n = 17/24; 70.8%), arterial blood gases (ABGs) were collected while the participant breathed room air, from which PaO_2_, arterial partial pressure of carbon dioxide (PaCO_2_), and arterial pH (pHa) were measured. In the whole cohort, SpO_2_ and estimated FiO_2_ were used to calculate the SpO_2_/FiO_2_ ratio. For the non-LTOT group, breathing room air SpO_2_/FiO_2_ was calculated assuming FiO_2_ = 0.209.

### Long-term oxygen therapy

Details about the past and current use of LTOT were collected considering: duration (in years) of LTOT prescription; use during daytime, night-time, and during exercise (Y/N); and O_2_ flow settings at rest (in L/min).

### Muscle oxidative capacity and arterial oxygen saturation

**Fig. 1:**
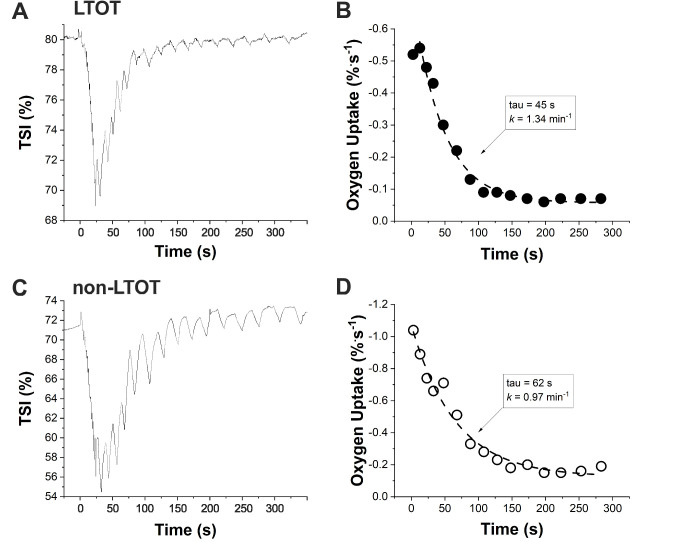
Representative examples of muscle oxygentation changes during intermittent arterial occlusions (left), and of muscle oxygen uptake recovery kinetics fitted for recovery rate constant (*k*; proportional to muscle oxidative capacity) (right). **A** and **B** Representative response for a LTOT COPD participant. **C** and **D** Representative response for a non-LTOT COPD participant

Medial *gastrocnemius* oxidative capacity was estimated from the muscle *k*, using NIRS combined with a series of fifteen arterial occlusions, as previously described (Adami et al. 2017, 2026). Briefly, this test relies on a property of activated muscle mitochondria, that the recovery rate constant (*k*, min^−1^; Fig. 1) of muscle oxygen consumption following contractions is directly proportional to muscle oxidative capacity. This concept has been validated in single isolated muscle fibers (Wust et al. 2013), and in comparison to muscle biopsy and ^31^P magnetic resonance spectroscopy in healthy people (Ryan et al. 2014, 2013). The test has strong reliability in patients with COPD (intraclass correlation coefficient (ICC) ≥ 0.88 (Adami et al. 2017; Adami and Rossiter 2018); current study ICC = 0.864).

For this protocol, the participant lies supine on a padded bed, and the NIRS probe (Portamon, Artinis, NL) is placed longitudinally along the belly of the *medial gastrocnemius*. The correct positioning of the NIRS probe is identified by palpation during few brief muscle contractions, and secured in place by an elastic bandage. A rapid-inflation pressure-cuff is placed few centimeters above the patella on the proximal thigh on the same leg as the NIRS probe, and then attached to an electronically-controlled rapid cuff-inflator (E20 and AG101, Hokanson, WA). The participant is first familiarized with sensation of rapid arterial occlusions, and also with the brief muscle contractions required in the protocol, which are plantar-flexion/relaxation against a manually-given light resistance at ~ 1 Hz frequency (hereafter referred to as muscle contractions). Then, after few minutes of rest to establish a stable baseline tissue saturation index (TSI), the test continues with a ~ 10–15 slight muscle contractions performed to increase mV̇O_2_, following which blood flow is occluded until a stable minimum TSI is reached or for 5 min (whichever occurs first). The cuff pressure is then released and the subsequent reactive reoxygenation monitored until resting baseline of NIRS signals is reestablished (typically ~ 3 min;Adami et al., 2017). This maneuver is used to define the individualized muscle TSI physiological normalization range. Two muscle oxidative capacity assessments follow, each consisting of: (1) ~ 10–15 s muscle contractions to increase mV̇O_2_ and desaturate the muscle to ~ 50% of the physiological normalization range; and (2) a series of rapid, intermittent arterial occlusions (5 occlusions for 5 s, 10 for 10 s, each separated by 5–20 s recovery). Each of the assessments last ~ 6 min and is separated by ~ 2 min of rest. The reported *k* value is the average from the two tests.

This protocol was administered to participants at both study sites (The Lundquist Institute and ICS Maugeri) using the same equipment and by technologists trained to ensure consistent protocol delivery. The data were analysed by a single reader. Further details about the device and protocol used are available in Adami et al. (Adami et al. 2026) for the *Muscle Health Study*, and protocol NCT04201548 for the RCT CE2288.

Arterial oxygen saturation (SpO_2_) was measured at rest, at the beginning of the NIRS test using fingertip pulse oximetry. The LTOT group performed the test using their prescribed resting oxygen flow settings.

### Statistical analysis

Data are presented as mean and standard deviation for continuous variables; and count, percentage, range (min–max), or median and interquartile range, for discrete variables. Chi-squared and Mann–Whitney U tests were used to determine group differences in discrete variables, and t-tests for independent samples to assess group differences for continuous variables. ANCOVA was used to determine any effect of LTOT on *k*, while adjusting for known covariates (FEV_1_%predicted, age, race (Adami et al. 2026).

Because we found that the LTOT group had a significantly lower body mass compared to non-LTOT, we also performed an exploratory analysis of the influence of BMI on *k*. We first performed a 1-way within-group ANOVA of differences in *k* stratified by BMI category for adults (CDC 2024). Secondly, the study population was dichotomized based on BMI (≤ 18.49 kg/m^2^ vs. ≥ 18.5 kg/m^2^) and a risk factor analysis for underweight vs. not underweight was explored in the full cohort with LTOT as a confounder.

Analyses were performed using SPSS v28 (IBM, Chicago, IL, USA), and graphical representations were produced using Prism 10 (GraphPad, San Diego, CA, USA). The significance level was set at 0.05.

## Results

A total of 52 individuals, previously enrolled in the two studies, qualified for our current analysis. Thirty (58%) participants from the *Muscle Health Study* met eligibility criteria and their previously reported data (Adami et al. 2026) were included in this analysis; 23 in the non-LTOT control group and 7 in the LTOT group. The other 22 participants were enrolled in the CE2288 trial; 6 in the non-LTOT group and 16 in the LTOT group.

Of the 52 participants, 18 (35%) were female, 9 (17%) were African American; 29 (56%) had very-severe COPD (GOLD 4) (Table 1). Twenty three (44%) participants were hypoxemic respiring room air and were prescribed LTOT treatment for median [IQR] time of 2 [1.0–4.4] years. Of these, 23 (100%) reported using supplemental oxygen at rest (flow median[IQR]: 1.0 [1.0–1.0] L/min, data available on n = 16/23) and during exercise (flow median[IQR]: 2.5 [2.0–3.0] L/min, data available on n = 23/23), and 22 (95.7%) reported use at night (flow median[IQR]: 1.0 [1.0–2.0] L/min, data available on n = 16/22).

**Table 1:** Characteristics of COPD patients with long term oxygen therapy (LTOT) compared to those without (non-LTOT)

	LTOT	non-LTOT	p-value
**Demographics and vital signs**			
N (%female)	23 (34.8)	29 (34.5)	0.982
Race (NHW/AA)(%AA)	21/2 (8.7)	22/8 (24.1)	0.209
Age (years)	70 ± 8	65 ± 9	0.060
Height (cm)	165 ± 11	168 ± 12	0.353
Weight (kg)	64 ± 16	76 ± 19	**0.019**
BMI (kg/m^2^)	23 ± 5	27 ± 6	**0.013**
Resting HR (min^−1^)	86 ± 13	77 ± 14	**0.028**
**Pulmonary function, clinical and smoking history**			
FEV_1_/FVC (%)	34.2 ± 6.6	40.0 ± 11.3	**0.023**
FEV_1_%pred (%)	28.6 ± 7.9	35.3 ± 6.4	**0.001**
FVC (%)	65.6 ± 15.5	71.3 ± 18.0	0.227
DL_CO_ (% pred)	30.8 ± 11.4	46.3 ± 12.3	** < 0.001**
Resting Nasal Cannula Oxygen Flow (L/min)	2.9 ± 1.1	–	–
Estimated FiO_2_%	31.4 ± 4.2	21.0 ± 1.3	** < 0.001**
Resting SpO_2_ (%)	94.6 ± 2.3^b^	96.1 ± 2.1^c^	**0.030**
Resting room air SpO_2_/FiO_2_	306.1 ± 39.3	453.5 ± 22.2	** < 0.001**
Resting room air PaO_2_ (mmHg) (n = 17)	57.0 ± 4.6	–	–
Resting room air PaCO_2_ (mmHg) (n = 17)	44.5 ± 7.6	–	–
Resting room air PaO_2_/FiO_2_ (n = 17)	271.6 ± 21.7	–	–
pHa (n = 17)	7.4 ± 0.0	–	–
Smoking history (ATS pack-years)	37.8 ± 11.6	51.4 ± 21.3	**0.016**
LTOT Duration (years [range])	3.2 [0.5–10]	0 [0–0]	–
Exercise performance			
6MWD (m)	264.6 ± 98.2^b^	371.3 ± 97.1	** < 0.001**
**Symptom questionnaires**			
mMRC	3.0 [3.0–3.0]	2.0 [1.5–3.0]	0.105
CAT total score	21.0 [17.0–23.5]	19.0 [9.0–23.0]	0.102
**Comorbidities**			
> 1 comorbidity (N(%))	20 (83)	26 (90)^a^	0.762
*Top five ranked conditions (in alphabetical order)*			
Diabetes mellitus	2 (8.7)	7 (24.1)	0.082
Gastroesophageal Reflux	3 (13.0)	4 (13.8)	0.815
Hypercholesterolemia	6 (26.1)	10 (34.5)	0.357
Hypertension	13 (56.5)	15 (51.7)	0.934
Osteoporosis	6 (26.1)	2 (6.9)	0.100

Data are mean ± SD, mean (min–max), count (%), or median [IQR]

Stastitically significant highlighted in bold

*COPD* chronic obstructive pulmonary disease; *NHW* non-Hispanic White; *AA* African American; *BMI* body mass index; *HR* heart rate; *FEV*_*1*_ forced expiratory volume in one second; *FVC* forced vital capacity; *DL*_*CO*_ lung diffusing capacity for carbon monoxide; *SpO*_*2*_ oxygen saturation; *FiO*_*2*_ inspiratory fraction of oxygen; *LTOT* long-term oxygen therapy; *PaO*_*2*_ oxygen arterial partial pressure; *PaCO*_*2*_ carbon dioxide arterial partial pressure; *6MWD* 6-min walking distance; *mMRC* modified Medical Research Council dyspnea Scale; *CAT* COPD Assessment Test

^a^n = 26 (comorbidity data was missing for 3 individuals)

^b^LTOT subjects used supplemental oxygen during these measurements

^c^Non-LTOT breathed room air

Table 1 shows a comparison of characteristics between groups. Relative to non-LTOT, the LTOT group had lower weight (p = 0.019) and BMI (p = 0.013), higher resting HR (p = 0.028), lower resting SpO_2_ (p = 0.030) despite oxygen supplementation, had worse pulmonary function (FEV_1_/FVC p = 0.023; FEV_1_%pred p = 0.001) and exercise performance (6MWD, p < 0.001), and lower DL_CO_ (p < 0.001). Furthermore, the LTOT group had a ~ 27% lower smoking history (p = 0.016). Dyspnea (mMRC p = 0.105) and COPD disease impact (CAT p = 0.102) were not different between groups. The LTOT group tended to be older (p = 0.060). Twenty (83%) of the LTOT group and 26 (90%) of the non-LTOT group had at least one concomitant chronic condition other than COPD. In the LTOT group, hypertension (56.5%), hypercholesterolemia (26.1%) and osteoporosis (26.1%) were the most prevalent comorbidities; in the non-LTOT group, prevalent comorbidities were hypertension (51.7%), hypercholesterolemia (34.5%), and diabetes mellitus (24.1%) (Table 1). By design, the LTOT group had lower SpO_2_/FiO_2_ compared to the non-LTOT group (p < 0.001; Table 1).

**Fig. 2:**
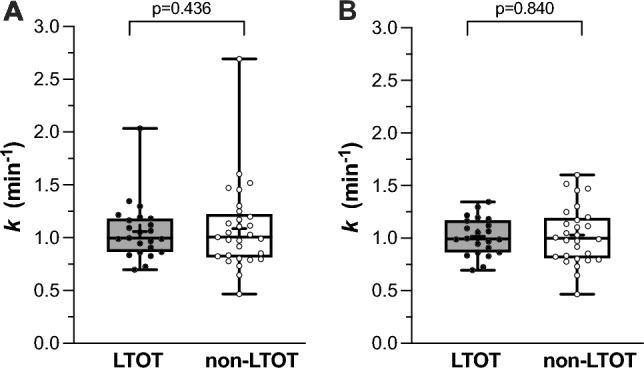
Comparison of muscle oxygen uptake recovery rate constant (*k*) between COPD patients with long-term oxygen therapy (LTOT) and those without (non-LTOT). **A** Full cohort. **B** After removing two outliers (see text for details). Raw data shown, unadjusted for covariates. P values are ANCOVA analysis results

Examples of the determination of muscle oxygen consumption recovery rate constant, *k,* for both groups are shown in Fig. 1, and group mean *k* in Fig. 2. After adjustment for known correlates (Adami et al. 2026), *k* was not different (p = 0.436; ANCOVA) between the LTOT (1.06 ± 0.07 min^−1^) and non-LTOT (1.09 ± 0.26 min^−1^) groups (Fig. 2A). We explored the influence of two outlying values that were  3SD above the mean, one from each group, but this did not change the finding that *k* was not different between LTOT and non-LTOT groups (p = 0.840; Fig. 2B).

Exploratory analysis of the influence of BMI on *k* found no within-group differences for the LTOT (p = 0.420) or the non-LTOT (p = 0.305) groups (1-way ANOVA stratified by BMI category) (Table 2). Using a risk factor analysis stratified by BMI (dichotomized), *k* remained similar between LTOT and non-LTOT groups (p = 0.435).

**Table 2:** Muscle oxygen consumption recovery rate constant (*k*) stratified by BMI, in patients with long term oxygen therapy (LTOT) and in those without (non-LTOT)

	LTOT (n = 23)	non-LTOT (n = 29)
**BMI category**		
Underweight		
n (%)	5 (21.7)	1 (3.4)
*k* (min^−1^)	1.18 ± 0.16	0.73
Normal weight		
n (%)	10 (43.5)	12 (41.4)
*k* (min^−1^)	1.09 ± 0.36	1.02 ± 0.27
Overweight		
n (%)	7 (30.4)	8 (27.6)
*k* (min^−1^)	0.92 ± 0.17	1.01 ± 0.23
Class 1 obesity		
n (%)	1 (4.3)	6 (20.7)
*k* (min^−1^)	1.18	1.22 ± 0.22
Class 2 obesity		
n (%)	0 (0)	2 (6.9)
*k* (min^−1^)	–	1.58 ± 1.57

Data are count (%), mean ± SD. BMI, body mass index; LTOT, long-term oxygen therapy

Underweight, $\le$ 18.49 kg/m^2^; Normal weight $\ge$ 18.5 to < 25 kg/m^2^; Overweight, $\ge$ 25 to < 30 kg/m^2^; Class 1 obesity $\ge$ 30 to < 35 kg/m^2^; Class 2 obesity $\ge$ 35 to < 40 kg/m^2^; (CDC 2024)

## Discussion

This retrospective study sought to identify whether chronic hypoxemia and LTOT were associated with lower muscle oxidative capacity in severely obstructed COPD patients. We hypothesized that severely obstructed COPD patients with chronic hypoxemia treated by LTOT would have lower muscle *k* compared to those without chronic hypoxemia or oxygen therapy. Contrary to our hypothesis, we found that locomotor muscle oxidative capacity assessed by NIRS was not different between COPD patients with or without LTOT (Fig. 2). Although non-LTOT patients had significantly greater BMI, which might affect NIRS measurements, we were unable to identify a significant influence of BMI on *k* in either group (Table 2).

Muscle oxidative capacity is a key mediator of endurance exercise capacity in health, and is increased by endurance exercise training. Muscle oxidative capacity is reduced in many patients with COPD, and low muscle oxidative capacity is associated with exercise intolerance and dyspnea symptoms (Maltais et al. 2000, 1996). Using the same NIRS-based assessment as used here, we previously showed that locomotor muscle mitochondrial oxidative capacity was ~ 34–37% lower in severe and very-severe COPD (GOLD 3–4) compared to ever smokers with similar smoking history but with normal spirometry (Adami et al. 2026), and that this impairment associates with reduced circulating di- or tri-acylglycerides (Li et al. 2021). Other studies demonstrate an association of impaired muscle oxidative capacity with increased free radical production, and systemic protein and DNA oxidation (de Batlle et al. 2010; Rodriguez et al. 2012). Chronic or intermittent hypoxemia is proposed as a potential mediator of low muscle oxidative capacity in COPD, but this hypotheses awaits further confirmation (Attaway et al. 2023, 2024; Kent et al. 2011; Couillard and Prefaut 2005; Gosker et al. 2000).

This study adds to the characterization of a major extrapulmonary manifestation in COPD by investigating the effects of long-term exposure to oxygen therapy on locomotor muscle mitochondrial oxidative capacity in, to our knowledge, the largest group of oxygen-treated people with severe pulmonary obstruction thus far. Using an in vivo assessment we found that muscle oxidative capacity was not different (p = 0.436) in severe COPD with chronic hypoxemia and LTOT, compared with normoxic severe COPD patients. This suggests that LTOT may contribute to protecting against loss of mitochondrial oxidative capacity that we would otherwise expect in severely hypoxemic patients. The premise of this suggestion is that the LTOT-treated COPD patients in this study had lower FEV_1_%pred and tended to be older than non-LTOT, each of which are associated with lower *k* (Adami et al. 2026). As such, we anticipated a lower *k* than in the LTOT group than we observed. It may be that there is a floor effect on muscle oxidative capacity, in that by the time patients progress to severe COPD muscle oxidative capacity does not fall any further. While the values of *k* in the severe COPD patients in this study are very low (~ 1 min^−1^ in both groups) compared to age-matched controls (~ 1.7 min^−1^; (Adami et al. 2026)), they remain greater than values observed in extreme immobilization, such as in invidividuals with spinal cord injury (~ 0.75 min^−1^) or motor-complete spinal cord injury (~ 0.5 min^−1^) (Erickson et al. 2013, 2017). Therefore, it seems plausible that further decline in muscle oxidative capacity is possible, even though we did not observe lower values here in LTOT-treated severe COPD patients; hence our speculation that LTOT may be protective against loss of mitochondrial oxidative capacity in severely hypoxemic COPD patients.

The precise mechanisms related to our findings in humans are currently unclear, but deserve further investigation. In murine models, intermittent or chronic hypoxia resulted in muscle oxidative dysfunction, disrupted supercomplex assembly, lower activity of respiratory complexes decreased mitochondrial fission and was associated with lower protein synthesis (Attaway et al. 2023). Therefore, based on our results, we speculate that protection against intermittent or chronic hypoxemia using LTOT may ameliorate some of these effects in humans. Alternatively, preserved muscle oxidative capacity in the LTOT group may reflect an indirect effect, such as increasing physical activity. While we did not measure physical activity in this study, we have previously shown that accelerometer-measured physical activity (steps per day and vector magnitude units) was not associated with muscle oxidative capacity in smokers with or without COPD (Adami et al. 2026). Therefore, we favor the hypothesis that LTOT has a direct protective effect on muscle mitochondrial function in chronically hypoxemic severe COPD patients, perhaps via the regulation of the reactive biology of oxygen.

Our findings are consistent with the improvement in muscle phosphate turnover observed in a small group of four COPD patients with chronic respiratory failure who underwent a *vastus lateralis* muscle biopsy after administration of 6-to-8 months of LTOT (Jakobsson and Jorfeldt 1995). Jakobsson and Jorfeldt (1995) showed that LTOT increased skeletal muscle PCr/(PCr + Cr) by 30%, reflective of an increased rate of mitochondrial oxidative phosphorylation in resting muscle. Our study expands those findings by evaluating a larger group of LTOT-treated COPD patients (n = 23) who had a longer exposure to oxygen therapy (~ 36 months vs. 6–8 months in the study of Jacobsson & Jorfeldt 1995). We also assessed a different locomotor muscle (*gastrocnemius* vs. *vastus lateralis*) and used an in vivo, non-invasive, method that is highly feasible, lower cost, faster and well tolerated by older individuals and people with chronic disease (Adami and Rossiter 2018).

In another study using MRS, Payen and colleagues (Payen et al. 1993) showed that exercise-induced increase in Pi/PCr and decrease intramuscular pH were less after acute administration of O_2_ in a group of seven stable COPD patients with chronic respiratory failure. Together these two previous studies and ours are consistent with a preserved muscle mitochondrial oxidative capacity by LTOT (our study and that of Jakobsson & Jorfeldt (Jakobsson and Jorfeldt 1995)), or acutely increased O_2_ supply (Payen et al. 1993), in COPD patients with chronic hypoxemia. It could be argued that the use of prescribed LTOT during NIRS testing could acutely compensate for mitochondrial dysfunction e.g., that *k* is influenced by greater O_2_ delivery rather than by a long-term, protective effect of LTOT. However, the *k* estimated in vivo using the NIRS-based protocol is deliberately conducted in non-oxygen limiting conditions (determined by maintaining the tissue saturation index (TSI) > 50% of the minimum value established during prolonged arterial occlusion). Under such conditions, *k* is correlated with maximal muscle O_2_ flux in fiber bundles (biopsy samples of *vastus lateralis* muscle), and is unaffected by convective or diffusive oxygen delivery (Adami et al. 2017; Pilotto et al. 2022). As such, our NIRS-based approach is not influenced by oxygen delivery and is therefore consistent with a muscle oxidative capacity in hypoxemic LTOT-treated severe and very-severe COPD patients that is not worse than normoxemic patients without LTOT treatment.

### Limitations

Our study has limitations that prevent us from fully describing the possible effects of LTOT on the management of hypoxemic COPD patients. First, ABGs data were not available for the non-LTOT group and several of the LTOT group, as such we cannot comprehensively describe the degree of resting hypoxemia in our cohort. To estimate the degree of pulmonary gas exchange impairment we used a surrogate index (SpO_2_/FiO_2_ ratio) where FiO₂ was estimated based on the administered oxygen flow rate by nasal cannula (Shapiro et al.^1982^). Second, the use of LTOT was self-reported and we do not know the degree of adherence to therapy, which possibly limits our ability to quantify the potential long-term protective effects of LTOT in this cohort. The available data for this retrospective study meant that we were not able to identify potential mechanisms by which LTOT is apparently protective. Although we excluded individuals who were participating in or had completed pulmonary rehabilitation exercise training within 18 months, we did not measure the physical activity of the participants, and therefore we do not know whether our results were influenced by differences in physical activity between groups. We did not investigate chronic hypoxemic COPD patients who were not receiving LTOT treatment (denial of treatment would be unethical) and did not have access to blood or muscle samples that may shed light on molecular mechanisms mediating benefit. Lastly, we were unable to detect a difference in *k* between groups, which risks a type II statistical error. Based on the effect size of *k* between groups measured in this study (Cohen’s d = 0.15) we estimate that 348 participants per group would be needed to determine with a power of 1 − β = 0.8 whether muscle oxidative capacity differred between those with and without LTOT.

## Conclusions

Contrary to our hypothesis, skeletal muscle oxidative capacity, measured using NIRS, was not different between hypoxemic severe and very-severe COPD patients with a long term history of oxygen supplementation therapy and severe and very-severe COPD patients who oxygenated well on room air. Because COPD patients with LTOT had lower FEV_1_%pred and were slightly older (known correlates of *k*), our findings suggest that LTOT may protect against loss of muscle oxidative function in COPD patients with chronic hypoxemia.

## Data Availability

Requests for de-identified participant data and study-related documents can be made through the corresponding author.

## Abbreviations

6MWD: 6-minute walk distance
ABGs: Arterial blood gases
ANCOVA: Analysis of covariance
ATS: American Thoracic Society
BMI: Body mass index
CAT: COPD Assessment Test
COPD: Chronic obstructive pulmonary disease
Cr: Creatine
DNA: Deoxyribonucleic acid
DL_CO_: Lung diffusing capacity for carbon monoxide
FEV_1_: Forced expiratory volume in one second
FEV_1_%pred: FEV_1_ percentage predicted
FiO₂: Inspired fraction of oxygen
FVC: Forced vital capacity
GOLD: Global initiative for chronic obstructive lung disease
HR: Heart rate
IQR: Interquartile range
*k*: Muscle oxygen consumption recovery rate constant, index of muscle oxidative capacity
LTOT: Long-term oxygen therapy
mMRC: modified Medical Research Council Dyspnea Scale
MRS: Magnetic resonance spectroscopy
NIRS: Near-infrared spectroscopy
PaCO_2_: Carbon dioxide arterial partial pressure
PaO_2_: Oxygen arterial partial pressure
pHa: Arterial pH
PCr: Phosphocreatine
PO_2_: Oxygen partial pressure
ROS: Reactive oxygen species
SpO_2_: Arterial oxygen saturation, estimated by pulse oximetry
TSI: Tissue saturation index
